# Driving Private Vehicles in Later Life: Necessity or Luxury?

**DOI:** 10.1093/geroni/igaa057.1505

**Published:** 2020-12-16

**Authors:** Moon Choi

**Affiliations:** KAIST, Daejeon, Taejon-jikhalsi, Republic of Korea

## Abstract

The automobile industry in South Korea has rapidly expanded for the last half century. The current generation of older adults is the first generation that has experienced mass car ownership and also faced challenges related to driving cessation due to geriatric syndromes. This study aimed to explore diversity in reasons for driving private vehicles among Korean older adults. Data came from a nationally representative survey on adults aged 65 years and older conducted in August, 2019 (N=1,500; women 57.1%). One out of four respondents (24.5%) reported driving, and the current drivers were categorized into four groups by primary reason for driving: (a) for convenience, (b) for caregiving, (c) for survival (due to the lack of public transport) and (d) for living (i.e. taxi or bus driver). The results showed that current drivers were more likely to be men, younger and more educated compared to non-drivers. Conspicuously, those who reported driving for their family members with mobility needs were the second largest group after those who reported driving for convenience such as for grocery shopping (34.6% and 43.6%, respectively). The results of multinomial logistic regression model showed that older age, living in rural areas and poorer self-rated health were associated with driving for survival as compared to driving for convenience. About the odds of driving for caregiving compared to driving for convenience were higher for those married and living with their spouse than for those not-married. The results imply the importance of considering diversity in developing public policies for older drivers.

